# Female Off-Farm Employment and Fertility Timing in Rural China

**DOI:** 10.3389/fpubh.2022.790436

**Published:** 2022-03-31

**Authors:** Zheng Shen, Zhao Zhao, Qisheng Ren, Mingwei Yan

**Affiliations:** ^1^School of Economics and Management, Zhejiang A&F University, Hangzhou, China; ^2^Zhejiang Province Key Cultivating Think Tank—Research Academy for Rural Revitalization of Zhejiang Province, Zhejiang A&F University, Hangzhou, China; ^3^School of Economics and Management, Nanjing Agricultural University, Nanjing, China

**Keywords:** off-farm employment, fertility timing, women, rural, China

## Abstract

**Background:**

Advanced maternal age is associated with fetal outcomes such as higher risks of birth defects and very low birth weight. Off-farm employment is an important factor in fertility transition in many developing countries. This study investigated the association between off-farm employment and fertility timing among Chinese rural women.

**Method:**

Using data from the China Labor-force Dynamics Survey (CLDS), we employed the ordinary least squares and instrumental variable approaches to estimate the effect of female off-farm employment on fertility timing decisions as well as the differences in the effect across groups.

**Results:**

The results show that off-farm employment participation is significantly associated with a later age at first birth, and the effect is stronger for women participating in wage employment than in off-farm self-employment. The delayed effects on fertility timing are also more pronounced for less-educated women and low-income families, implying a heterogeneous effect in terms of women's socioeconomic status.

**Conclusion:**

Studies of the relationship between women's off-farm employment and fertility timing in rural areas of developing countries remain limited. This study provides important insights on this topic, and it lends support to efforts to design effective policies and practices to facilitate female employment, childbearing, and health promotion.

## Introduction

The rapid industrialization and urbanization in many developing countries in recent decades has resulted in more off-farm work for farmers and rural women (1). These expanding employment opportunities play an important role in determining rural women's fertility decisions or behaviors, including the timing of fertility. Women's fertility timing is believed to be associated with fetal outcomes and mother's health status; pregnancy at an early age or at an advanced maternal age may increase the risk of poor physical and mental health. While numerous studies of the relationship between female labor supply and fertility have been conducted for industrialized countries and urban areas (2–6), the effect of off-farm employment on fertility timing among rural women in developing countries has received less attention. Given that maternal care services are insufficient and family support systems are weak in developing regions, investigating the association of women's employment and fertility timing matter for polices to promote maternal and child health.

China is one example of a rapidly growing country that has seen female employment in the non-agricultural sector (industry and services) increase sharply over the last few decades, from about 43.3% in 1991 to 75.8% in 2018 (7). This change is particularly evident for rural women aged 16–30 years, among whom more than seven out of 10 engage in off-farm labor (8). The high prevalence of female off-farm employment in China reflects rural women's socioeconomic transition from a pattern of smallholder families to a working pattern with a higher opportunity cost for raising a child. As such, women's fertility decisions are likely to change from the traditional desire for more children and early childbearing to a modern fertility pattern that demands fewer children and tends to have children at an advanced maternal age.

Recent studies on fertility effects have been examined using several empirical methods. Based on a sample of 1,800 women of childbearing age in the United States, Shreffler (9) used logistic regression to determine the associations between professional work characteristics and the postponement of childbearing. The results showed that women with more professional jobs were more likely to postpone childbearing. To control for the problem of unobserved heterogeneity, Alam and Pörtner (10) employed a fixed effect model for the impact of income shocks on pregnancies and births in Tanzania. The estimates indicated a significant decrease in the likelihood of pregnancy and birth after a crop loss for rural families. Li et al. (11) estimated the effect of gender egalitarian attitudes on fertility desires using a multilevel logistic model, and they found that such attitudes are associated with lower fertility desires for the full sample while the negative effect is stronger for women than for men. Another stream of the literature tried to account for the possible endogeneity of employment using the instrumental variable approach. Using data from the China Health and Nutrition Survey in 2006, Fang et al. (12) found female employment to be endogenous, with the instrumental variable estimation showing a significant fertility-reducing effect of employment participation. Shen et al. (7) used parental education and parental work status as instruments for off-farm employment. The employment participation was found to reduce women's preference for the number of sons.

In terms of the effect of employment on fertility timing, our paper contributes to the existing research by taking into account endogeneity that might bias the estimates. To address this issue, we applied multiple methods (including instrumental variable, propensity score matching, and fixed effects) to data from the China Labor-force Dynamics Survey (CLDS), which not only provides information on people's employment history and fertility records, but also enabled us to construct a plausible instrument for employment behavior among rural residents. We not only examined differences in the relationship between off-farm employment and fertility timing in terms of self-employment and wage employment, but we also discuss how the effects differ by women's age, education, and household income. This allows us to reveal differences in the effects across groups and to understand the heterogeneity in the labor market.

The remainder of this paper is organized as follows. The Section Literature Review presents previous literature on female employment and fertility. The Section Materials and Methods describes the data, measures, and empirical methods. The Section Results presents the descriptive statistics, main regression results, and subgroup analyses. Finally, in the Section Discussion and Conclusions, we discuss the implications and limitations.

## Literature Review

Research in economics has long discussed the theories of decisions related to childbearing. According to the Malthusian theory of population growth, fertility should increase as income increases under the assumption that moral restraints (e.g., abstinence and delayed marriage) against fertility would be weakened by higher income (13). Neoclassical economic theory predicts a positive relationship between fertility and income when children are characterized as a normal good in a simple model. However, most empirical evidence is at odds with this prediction and shows a decrease in fertility rates as countries become wealthier. Two main factors may account for this fact within a neoclassical model. First, if a tradeoff between quantity and quality of children is introduced, income increases will reduce fertility if income elasticity for the number of children is smaller than income elasticity for the quality of children (14–16). Second, child-rearing requires an intense amount of a mother's time, so higher female earnings increase the marginal cost of time allocated to childcare, decreasing the demand for children (16–18).

Theoretically, there are several possible mechanisms for how off-farm employment affects women's fertility timing. First, women's off-farm employment might be positively associated with postponing fertility, as it leads to a higher opportunity cost of childbearing among employed women compared to women working on a farm. This is especially the case in China, where urbanization can worsen mothers' dual burden of paid and unpaid “second shift” work (19). More importantly, employed women may worry about the “mommy track” and indeed often experience a “motherhood wage penalty,” meaning that having children will interrupt career advancement or reduce future wages (9, 20). Postponing fertility may be a successful strategy against the motherhood wage penalty.

Second, women participating in the off-farm labor market need to adapt to a different environment; this includes the adjustment to the socioeconomic, social network, and gender norms of the living and working environment as well as to the economic constraints and opportunities that they face as a result of employment (21, 22). This adaptation process has a two-fold effect on women's motivation to have children. On the one hand, through off-farm employment, women may accept more modern norms and shift from traditional to modern attitudes toward fertility, which could weaken their son preference and desired number of children. On the other hand, newly off-farm workers may experience a state of stress and uncertainty when working and living in an unfamiliar environment, and they prefer to have children only after adapting to their new life environment (23). Hence, the adaptation effect of employment participation may lead a woman to delay childbearing.

Third, off-farm employment and associated economic uncertainty might affect women's fertility timing decisions. A higher risk of future unemployment and income uncertainty are more prevalent among workers engaged in low-skill work, temporary jobs, and lower job status. This is especially true for off-farm workers in China, where rural people engaging in non-agricultural jobs mainly work in lower occupational status positions and low-skilled jobs. It might be worthwhile for employed women to postpone their childbearing to a later period, because parents can avoid entering parenthood and incurring its associated irreversible costs in a poor situation when having children is not optimal (6). To reduce some of the uncertainty, women who participate in off-farm work tend to delay childbearing.

Previous studies have investigated the links between female employment and fertility at the individual level. For instance, Schultz and Zeng (24) found that the shift in the labor force from agricultural activity to industrial production is associated with a decline in fertility: a drop in agricultural employment from 90 to 60%, replaced by industrial employment, is associated with an additional 0.06 decline in children in the younger sample of women and an additional 0.24 decline in the older sample. Kalwij (2) found that, in the Netherlands, employed women have fewer children than non-employed women do. Using data from the United States between 1979 and 1994, Budig (4) found that both part- and full-time employment reduced the likelihood of pregnancy for all ethnic groups. Auer and Danzer (6) obtained similar results in the case of Germany, where women with fixed-term employment in the labor market tended to reduce the number of children in the first 10 years after graduation. Fang et al. (12) used data from the China Health and Nutrition Survey in 2006 to show that female employment was associated with a 0.35 reduction in the preferred number of children and a 0.50 reduction in the actual number. The empirical evidence on Sub-Saharan Africa also shows a fertility-reducing effect of female employment. There was a 25% reduction in the number of children for employed women in Senegal, and the effect was larger for the illiterate population (25). Previous research showed a negative relationship between women's employment and the desired and actual number of children. Little is known, however, about rural women's fertility timing responses to their off-farm employment behaviors. The present article aims to fill this research gap.

## Materials and Methods

### Data

The data for this study come from the 2014, 2016, and 2018 waves of the CLDS, a nationally representative survey conducted by the Center for Social Survey at Sun Yat-sen University. The CLDS uses multi-stage, stratified probability proportional-to-size (PPS) sampling to draw data on ~400 communities from 29 provinces in mainland China. This dataset contains a wide range of information on demographic and family characteristics, employment status, and a variety of survey questions about childbearing history for women. The CLDS was undertaken according to the guidelines laid down in the Declaration of Helsinki, and all of the procedures involving human participants were approved by the Institutional Review Board of Sun Yat-sen University. Written informed consent was obtained from all of the respondents.

This study examined the effects of employment on fertility timing among Chinese rural women. To realize this goal, the sample was restricted to individuals who had an agricultural *Hukou*—that is, the household registration system that identifies whether a respondent is a rural citizen (8). This study retained women in the age range from 20 to 49 years because this represents the majority of the childbearing population, and those women who have never had a child were excluded from the study sample. This study excludes the observations from those who appeared not to be working (e.g., unemployed, at school or in training, or on maternity leave) to ensure that the sample would reflect the fertility timing decisions of labor force participants. Our final study sample consisted of 6,188 individuals, of whom 3,239 were engaged in off-farm employment and 2,949 engaged in household farm work.

### Measures

#### Dependent Variable

Fertility timing was measured by women's age at their first birth, an explicit measure of timing as a dependent variable commonly used in regression analyses in various forms (26). In the CLDS, respondents were asked, “How old were you when your first child was born?” Responses to this question were used as the dependent variable to reflect the fertility timing among the rural women surveyed. It is a continuous variable, and higher values indicate that women have a later age at fertility.

#### Key Independent Variable

The key independent variable in our study is off-farm employment, which is a dichotomous variable reflecting whether women participated in full-time employment in the non-agricultural sector for the first job in their lives. This variable was determined based on responses to following questions. First, respondents were asked, “What is your current working status?” If they responded that they were engaged in any work activities with labor income, they were asked a further three questions: “Is this a full-time job with at least 35 h of work each week?” “Which sector type does your job belong to?” “Is this your first job in your life?” The variable for off-farm employment equals one if the respondent's first full-time employment belonged to non-agricultural sectors, and zero if the respondent's first job involved in agriculture. Time and financial constraints faced by women may vary by employment status, which may result in heterogeneous effects on fertility timing across different work characteristics. To investigate this heterogeneity, employment status was divided into three categories: household farm work (the reference group), off-farm self-employment, and off-farm wage employment. Women participating in off-farm wage employment were expected to be more likely to postpone their age at first birth.

#### Control Variables

To account for potential confounding factors that may be both correlated with women's employment and fertility decisions, we controlled for a set of variables related to individual, family, and community characteristics. Demographic variables included age, education, religious beliefs, and ethnic identity. Among them, age is a continuous variable that may reflect the women's attitudes toward fertility at different stages of life. Education, measured as the respondent's years of formal schooling completed, was expected to be positively correlated with a higher maternal age because women with a high level of education may choose social patterns less likely to lead to early marriage (27). Religion is an important factor in women's attitudes motivating childbearing (28), and it was measured by respondents' religious affiliation. Because China is a large and diverse country, we controlled for respondents' ethnic identity using a dummy variable coded as 1 if the respondent was from an ethnic minority group and 0 if the respondent identified as Han (7). For health status, two measures were used to reflect the self-rated and objective health of women: a dummy variable coded as 1 if the respondent reported her health status in good condition, and a dummy variable coded as 1 if the respondent reported having ever been hospitalized due to injury or illness. Both self-rated and objective measures have been found to be strong predictors of fertility intentions (29).

Previous studies have suggested that a variety of social security programs may alter financial incentives regarding fertility (30, 31). We captured this effect with three measures: a maternity insurance dummy (1 if the respondent was covered by maternity insurance), a health insurance dummy (1 if the respondent was covered by any type of social health insurance), and a public pension dummy (1 if the respondent participated in the governmental pension scheme). Happiness was measured using a single question on subjective wellbeing, ranging from “1 = very unhappy” to “5 = very happy.” We also controlled for the effects of desired number of children and household income on fertility timing. The regression models also included community-level characteristics such as childcare facilities and health clinics, which reflect infrastructure conditions and access to public services.

### Empirical Strategies

We first examined the association between off-farm employment and fertility timing using the ordinary least squares (OLS) estimation method. The OLS specification takes the following form:


(1) $$\begin{array}{r} Y_{i}=\beta_{0}+\beta_{1}OFM_{i}+\beta_{2}X_{i}+\mu_{i}, \end{array}$$

where *Y*_*i*_ is the dependent variable related to the outcomes of women's fertility timing (the age at first birth). The main variable of interest, *OFM*_*i*_, is off-farm employment, which is specified as a dummy variable taking the value one if individual *i* participated in non-farm labor market for her first full-time job. *X*_*i*_ is a vector of control variables.

In OLS models, we acknowledge that the explanatory variable of interest, off-farm employment, may be endogenous due to individual unobserved heterogeneity, such as ability and risk preference, which may affect both off-farm participation and fertility decisions. These unobserved factors would cause our estimates of employment and fertility effects to be correlated with the error term, resulting in bias. To address this concern and to estimate the causal effects, an instrumental variables (IV) approach is employed. The IV identification strategy requires that our instruments are not correlated with unobserved characteristics while they strongly explain the endogenous variable. We carried out a two-stage least squares (2SLS) procedure using the number of enterprises and distance to the nearest county (km) as instruments for off-farm work participation. The rationale for our IV selection was that these instruments are highly correlated with convenience to access to information and labor markets and the cost of participation in employment, while they are not influenced by individual fertility behaviors.

Additionally, the OLS estimates might also be biased due to the potential problem of sample selection. We employed a propensity score matching (PSM) technique to address this bias and estimate the average treatment effect on the treated (ATT) (32). First, a binary logistic regression model was used to estimate the propensity scores by including explanatory variables that might influence the likelihood of women's employment and fertility timing. These explanatory variables were the same as the covariates used in the aforementioned OLS regression. Second, the estimated scores were used to match women from the off-farm (treatment) and farm (control) groups within the common support region using different matching techniques, and the balanced properties were tested. Finally, we used the matched samples to estimate the treatment effects of off-farm employment on rural women's fertility timing.

## Results

### Descriptive Statistics

The means of the variables used in this study are presented in Table 1. In the full sample, the average age at first birth is 23.56. The mean value of individual age and years of formal education are 39.56 and 8.422 years, respectively. The percentage of women who have a religious affiliation is 11.8 and 9.1% of the sample were from an ethnic minority group. In addition, 61.2% of women reported having a good health condition, and 6.6% had been hospitalized due to injury or illness at some point. In terms of social security, 91.4% of the sample has social health insurance, and 58.2% participate in a public pension scheme, while only 9.1% of women are covered by maternity insurance. The average desired number of children is 2.065, suggesting that rural women tend to have a fertility desire of at least two children. The mean value of women's subjective wellbeing is 3.731, and the logarithm of annual average household income is 10.51. Above 50% of communities have a childcare facility and health clinic for local residents.

**Table 1:** Definition of variables and mean for the full sample and by working status.

**Variables**	**Definition of variables**	**Full sample**	**Household farm work**	**Off-farm employment**
Fertility timing	Women's age at first birth	23.56	23.12	23.96
Age	Age at time of survey	39.56	41.91	37.42
Education	Respondent's years of formal education	8.422	7.323	9.421
Religion	One or more religious affiliations equals 1; no religious affiliation equals 0	0.118	0.102	0.133
Ethnic minority	Ethnic minority group status (1 = minority groups, 0 = Han identity)	0.091	0.149	0.036
Good health	Self-reported health status (1 = good, 0 = fair or poor)	0.612	0.538	0.678
Hospitalization	Having ever been hospitalized due to injury or illness (1 = yes, 0 = no)	0.066	0.071	0.061
Maternity insurance	Maternity insurance coverage equals 1, none is 0	0.091	0.005	0.169
Health insurance	Social health insurance coverage equals 1, none is 0	0.914	0.927	0.903
Public pension	Participation in any governmental pension scheme equals 1, none is 0	0.582	0.609	0.558
Happiness	Women's subjective wellbeing	3.731	3.644	3.809
Desired number of children	Women's desired number of children	2.065	2.117	2.017
Household income	Annual household total income per capita (Yuan, log)	10.51	10.11	10.87
Childcare facilities	Whether the community had childcare facilities (1 = yes, 0 = no)	0.555	0.450	0.651
Health clinics	Whether the community had health clinics (1 = yes, 0 = no)	0.861	0.855	0.866
Observations		6,188	2,949	3,239

Table 1 also provides the means of the variables by working group. The results indicate that there is a difference in fertility timing between household farm work and off-farm employment. Specifically, women with an off-farm job tend to postpone their fertility decisions (23.956) later than those engaging in farm work activities (23.124). In addition to fertility timing, other covariates differ by working status as well. For example, respondents who participate in the off-farm labor market are younger (37.42 vs. 41.91 years old) and more highly educated (9.42 vs. 7.32 years of education), and the proportions of women having a public pension scheme and ethnic minority are smaller for off-farm workers (3.6 vs. 14.9% and 55.8 vs. 60.9%). Moreover, women participating in off-farm work reported being better health (67.8 vs. 53.8%), having higher maternity insurance coverage (16.9 vs. 0.5%), and being more likely to have a religious affiliation (13.3 vs. 10.2%), while they showed a lower rate of hospitalization (6.1 vs. 7.1%) and desired fewer children (2.017 vs. 2.117).

### Effects of Off-Farm Employment on Fertility Timing

Table 2 presents the estimates for the effects of women's off-farm employment on fertility timing. The result of the OLS model shows that the marginal effect of off-farm employment is 0.441, which is significantly different from zero, suggesting that, compared to women working on the farm, those who participated in off-farm employment are more likely to postpone childbearing. This is consistent with the results from the descriptive statistics, as shown in Table 1, where women's age at first birth in the off-farm employment group is higher than that of the household farm work group. The second column shows the results for the 2SLS regression: women's participation in off-farm employment leads to an increase in maternal age by 1.341, significant at the 5% level. Compared to the OLS results, the 2SLS point estimates show a larger magnitude for the effect of employment on fertility timing. One possible reason is that the IV method for estimation of local average treatment effects (LATE) may reflect those who benefit more from employment are more likely to self-select into the labor market. Nevertheless, as seen from Appendix Table 2, the instruments (i.e., number of enterprises and distance to the nearest county) are relevant, because the coefficients on the instruments for the first stage regression of 2SLS are both significant at the 1% level. Moreover, the first stage F-statistic for IV (64.28) is higher than 10 and the critical value of 19.93 for 10% IV size, indicating that the instruments are not weak.

**Table 2:** Estimates for the association between off-farm employment and fertility timing.

	**Fertility timing**	
**Variables**	**OLS**	**2SLS**
Off-farm employment	0.441^***^	1.341^**^
	(0.087)	(0.600)
Age	0.041^***^	0.050^***^
	(0.005)	(0.008)
Education	0.213^***^	0.173^***^
	(0.017)	(0.032)
Religion	−0.063	−0.124
	(0.106)	(0.114)
Ethnic minority	−0.273^**^	−0.088
	(0.131)	(0.179)
Good health	0.059	0.031
	(0.078)	(0.082)
Hospitalization	0.266^*^	0.292^*^
	(0.153)	(0.153)
Maternity insurance	0.798^***^	0.595^***^
	(0.141)	(0.193)
Health insurance	−0.083	−0.030
	(0.132)	(0.138)
Public pension	−0.151^*^	−0.082
	(0.078)	(0.091)
Happiness	−0.145^***^	−0.138^***^
	(0.042)	(0.043)
Desired number of children	0.040	0.032
	(0.061)	(0.062)
Household income	−0.056	−0.151^**^
	(0.041)	(0.076)
Childcare facilities	0.114	−0.007
	(0.075)	(0.108)
Health clinics	0.068	0.128
	(0.109)	(0.116)
Constant	20.902^***^	21.367^***^
	(0.529)	(0.623)
First stage F-statistic for IV		64.28
*p-*value of Hansen J-statistic		0.742
Observations	6,188	6,188

*** *,*

** *, and*

* *Indicate significance at the 1, 5, and 10% levels, respectively. Robust standard errors are presented in parentheses*.

We also applied the PSM approach to overcome the selection problem. Kernel densities were plotted in Appendix Figure 1 to examine the distributions of propensity scores across farm and off-farm groups, which appeared reasonably similar after Kernel matching. The balance tests of the characteristics between farm and off-farm work before and after matching are also presented in Appendix Table 1. Significant differences appeared for some characteristics between farm and off-farm groups before matching. For example, older and less-educated women and those who are in poor health or from an ethnic minority group were less likely to participate in off-farm employment. After matching, most of the covariates are no longer significant between the two groups (*p* < 0.05), and the standardized bias across covariates is close to zero, suggesting that sample selection bias is largely corrected and that the matching estimates have high validity. Table 3 reports the PSM estimation results for three types of matching, including nearest neighbor matching (*k* = 1, *k* = 2, and *k* = 3), radius matching (*r* = 0.1, *r* = 0.01, and *r* = 0.001), and kernel matching (Epanechnikov, normal, and biweight). The point estimates of ATT show that off-farm employment is significantly associated with an increased age at first birth by 0.38–0.48 cross different matches, which are similar to the results in the OLS regression models but smaller than those from the 2SLS models.

**Table 3:** PSM estimates for the effects of off-farm employment on fertility timing.

	**Fertility timing**		
**Nearest neighbor matching**	***k*** **= 1**	***k*** **= 2**	***k*** **= 3**
ATT	0.445^***^	0.453^***^	0.474^***^
	(0.168)	(0.152)	(0.152)
Observations	5,920	5,920	5,920
*Radius matching*	*r* = 0.05	*r* = 0.01	*r* = 0.001
ATT	0.387^***^	0.397^***^	0.469^***^
	(0.132)	(0.149)	(0.125)
Observations	5,920	5,920	5,486
*Kernel matching*	Epanechnikov	Normal	Biweight
ATT	0.404^***^	0.386^***^	0.407^***^
	(0.151)	(0.145)	(0.151)
Observations	5,920	5,920	5,920

*** , ^**^ and ^*^Indicate significance at the 1, 5, and 10% levels, respectively. Standard errors are presented in parentheses.

One more threat to the estimated results may come from potential bias related to unobserved heterogeneity. We addressed this concern by employing the fixed effect (FE) regression model. Results are presented in Table 4. In column (1), the regression model only includes year fixed effects and provides similar results to the OLS and PSM estimations. When province fixed effects are included, as shown in column (2), the magnitude of the delayed effect on timing of fertility appears to be smaller, with an estimated coefficient of 0.225 at a significance level of 5%. In column (3), after controlling for county fixed effects, the size of off-farm employment coefficient continues to decline and is no longer significant. This suggests that the effect of off-farm work participation on women's fertility timing may be largely accounted for by regional heterogeneity in the relationship between employment and fertility.

**Table 4:** Fixed-effect estimates for the impact of off-farm employment on fertility timing.

	**Fertility timing**		
**Variables**	**(1)**	**(2)**	**(3)**
Off-farm employment	0.452^***^	0.225^**^	0.097
	(0.087)	(0.094)	(0.109)
Year FE	Yes	Yes	Yes
Province FE	No	Yes	No
County FE	No	No	Yes
Observations	6,188	6,188	6,188

*** *,*

** , and ^*^Indicate significance at the 1, 5, and 10% levels, respectively. Robust standard errors are presented in parentheses. Control variables are included in all regression models.

*FE, fixed effects*.

### Subgroup Analyses

We further investigated whether the link between off-farm employment and fertility timing differs across important individual characteristics. Regression analyses were performed based on the sample stratified by education (middle school or less and high school or more), employment status (off-farm self-employment and wage employment), and household income (low and high group divided by the mean of total household income). Table 5 reports the OLS and 2SLS estimates.

**Table 5:** Results by education, employment status and household income.

**Panel A: By education**	**Middle school or less**		**High school or more**	
	**OLS**	**2SLS**	**OLS**	**2SLS**
	**(1)**	**(2)**	**(3)**	**(4)**
Off-farm employment	0.505^***^	1.829^***^	0.217	−4.253
	(0.092)	(0.631)	(0.280)	(5.516)
Observations	5,176	5,176	1,012	1,012
**Panel B: By employment status**	**Off-farm self-employment**		**Wage employment**	
	**OLS**	**2SLS**	**OLS**	**2SLS**
	**(5)**	**(6)**	**(7)**	**(8)**
Off-farm employment	0.384^***^	1.881	0.472^***^	1.443^**^
	(0.125)	(1.587)	(0.095)	(0.618)
Observations	3,837	3,837	5,300	5,300
**Panel C: By household income**	**Low income**		**High income**	
	**OLS**	**2SLS**	**OLS**	**2SLS**
	**(9)**	**(10)**	**(11)**	**(12)**
Off-farm employment	0.546^***^	1.997^**^	0.360^***^	0.639
	(0.120)	(0.835)	(0.126)	(0.969)
Observations	3,294	3,294	2,894	2,894

*** *,*

** , and ^*^Indicate significance at the 1, 5, and 10% levels, respectively. Robust standard errors are presented in parentheses. Control variables are included in all regression models.

In Panel A, off-farm employment associated with fertility timing is separately estimated by educational attainments. Women with an education level of middle school or less postpone their fertility by 0.5–1.83 years when they participate in off-farm work, while there are no significant effects for women with a high school education or more. The effects on fertility timing appear to be driven by participation in wage employment, as demonstrated in Panel B. Compared to women engaging in farm work, off-farm wage employment is significantly associated with later childbearing in both cases of OLS and 2SLS. For women working in off-farm self-employment, the effect size appears to be smaller and only significant in the OLS estimation. Panel C presents the estimates stratified by income group and suggests that off-farm employment has a heterogeneous effect on fertility timing between low- and high-income groups. Estimates in the OLS and 2SLS models show that employed women in low-income families postpone the timing of fertility by 0.5–2.0 years. In contrast, the coefficient of off-farm employment is smaller for women in high-income families and not statistically significant in the 2SLS estimation.

## Discussion and Conclusions

Using Chinese individual-level microdata, we present an empirical analysis for the association of women's participation in off-farm employment with their fertility timing. The results indicate that off-farm employment is positively associated with rural women's age at first birth. Our findings support the view of the theories of fertility timing on the role incompatibility between employment and childbearing. If women have reason to believe that the timing of childbearing would interrupt their career advancement or employment prospects, they may postpone the birth of a first child. Previous research has indicated that when rural women participate in off-farm labor market, particularly in a developing society, it leads to a significantly negative effect on son preferences, fertility desires, and actual number of children (7, 12, 25). This study further confirms that, in addition to fertility-reducing effects, female non-farm employment is also associated with later age at childbearing. While off-farm employment is important for poverty reduction and life improvement, the non-income effects of the off-farm labor market, such as fertility decisions, should not be ignored either. Family policies to reduce the conflicts between family and work in rural areas should be considered.

We found that the effects of off-farm employment on delayed fertility is weaker among off-farm self-employed women and stronger among wage-employed women. This may be due to the differences in work characteristics between off-farm self-employment and wage employment. It has been argued that off-farm self-employment is related to greater autonomy and flexibility, which could allow women to flexibly combine childcare and labor force participation (33). In contrast, when working in off-farm wage employment, the arrangements of working hours are less autonomous and flexible, which might lead to a higher level of incompatibility between maternal and job roles (7). Additionally, self-employed households are more likely to prefer more children to raise the probability that an inside family member will be a good match for continuing the family business (34). In fact, the characteristics of self-employment, such as flexibility and family inheritance, are very similar to those of peasant farming operations in rural China; indeed, both of them need more laborers in household production, so parents prefer larger families and have children earlier.

Our paper also shows that the association between off-farm employment and fertility timing differed in terms of education attainments and household income. A significant effect is found solely for those less-educated women, where age at first birth is increased by 0.2–0.3 years. It is difficult to interpret precisely what might cause this difference between educational groups. They are potentially related to the differences in the occupational expectations placed on less- and higher-educated people in their respective jobs. When less-educated women participate in the off-farm labor market, they might place a greater value on their jobs because they fear that childbearing will cause a future wage penalty or even dismissal. As such, having children is likely to put less-educated women in an even more unfavorable situation. The positive effect on delayed fertility is stronger for employed women in low-income households compared to women in high-income households. This is likely due to the fact that employed women in households with a high level of income could cope with increased economic uncertainty, while poorer women are more vulnerable to increased economic hardship and might be disadvantaged in the labor market when having children during an economic recession. It might therefore be worthwhile for women in low-income families to focus more on employment than on fertility before the uncertainty is addressed.

Our results in this study are specific for the case of rural China. The association between off-farm employment and fertility timing may suffer from measurement error due to economic conditions and cultural background, which warrants caution in applying our results to other countries and regions. Although we can conclude that off-farm employment is associated with delayed fertility among Chinese rural women—and additional methods, like 2SLS and PSM estimation, support our findings—the causal impacts remain uncertain, as possible unobserved heterogeneity may confound the estimates. Additional studies with other data sources and approaches will help to further strengthen our findings. Due to data limitations, we were unable to systematically investigate the possible mechanism through which off-farm work may affect women's fertility timing decisions, and more research is needed to investigate this.

Despite the limitations, this study provides important insights for the relationship between women's labor participation and fertility timing in China, and it has several implications for policy makers. First, as the number of rural women engaging in non-farm jobs continues to rise, family-based development plans to support female employment and childbearing are necessary. If the government needs to promote fertility while maintaining women's high levels of work engagement, family policies should consider variations among individual age and education and household income. Second, given that advancing maternal age might be adversely associated with mother's health and fetal outcomes, it is important guide rural women to choose to have children at an age when it is most suitable both psychologically and physiologically. Finally, we need to improve the social support system for child care, such as employer-sponsored programs, community service facilities, and male participation in child-care activities.

## Data Availability Statement

The data underlying the results presented in the study are available from the China Labor-force Dynamics Survey (CLDS). Anyone can access through application with the Center for Social Survey at Sun Yat-sen University (http://isg.sysu.edu.cn/node/353).

## Author Contributions

ZS and ZZ: conceptualization. ZS and MY: methodology. ZS, QR, and MY: validation. ZS, ZZ, and QR: formal analyses. ZS, ZZ, and MY: investigation. ZS, ZZ, QR, and MY: writing—original draft preparation. All authors contributed to the article and approved the submitted version.

## Funding

This research was funded by the National Natural Science Foundation of China (Grant Number: 71903177). Zhejiang University Student Science and Technology Innovation Activity Plan (New Seedling Talent Plan Subsidy Project, 2021R412017). National-Level College Students Innovative Entrepreneurial Training Program of Zhejiang A&F University (202110341048).

## Conflict of Interest

The authors declare that the research was conducted in the absence of any commercial or financial relationships that could be construed as a potential conflict of interest.

## Publisher's Note

All claims expressed in this article are solely those of the authors and do not necessarily represent those of their affiliated organizations, or those of the publisher, the editors and the reviewers. Any product that may be evaluated in this article, or claim that may be made by its manufacturer, is not guaranteed or endorsed by the publisher.
