# Supplementary material for: Female Off-Farm Employment and Fertility Timing in Rural China
Source: Front Public Health. 2022 Mar 31;10:790436. doi: 10.3389/fpubh.2022.790436 (PMC9009253; doi:10.3389/fpubh.2022.790436)
Supplement: Supplementary file 1 [file Data_Sheet_1.pdf]

Appendix

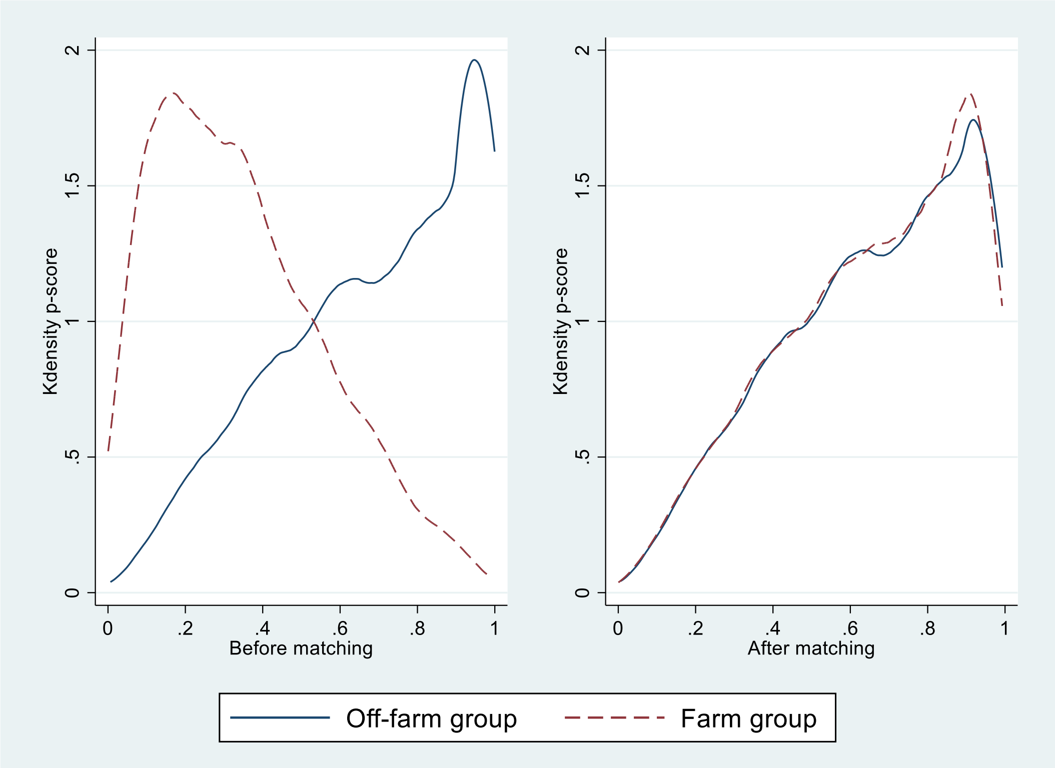

Figure A1 Density plots of propensity scores before and after Kernel matching

Table A1 Covariate balance testing for propensity score matching

Variable	Unmatche[illegible] Matched	Mean		% Bias	% Reduct |bias|	*t*-test	
		Treated	Control			*t*-statistics	*p*>|t|
Age	U	37.419	41.911	−64		−25.07	0.000
	M	38.458	38.117	4.9	92.4	1.59	0.111
Education	U	9.421	7.324	93.8		36.51	0.000
	M	8.766	8.668	4.4	95.3	1.61	0.108
Religion	U	0.133	0.102	9.5		3.73	0.000
	M	0.129	0.135	−1.6	82.9	−0.55	0.580
Ethnic minority	U	0.036	0.149	−39.7		−15.8	0.000
	M	0.043	0.045	−1	97.6	−0.48	0.633
Good health	U	0.678	0.538	28.9		11.39	0.000
	M	0.658	0.683	−5.3	81.7	−1.94	0.053
Hospitalization	U	0.061	0.071	−4.2		−1.65	0.100
	M	0.056	0.060	−1.8	56.7	−0.68	0.494
Maternity insurance	U	0.170	0.005	61.1		23.49	0.000
	M	0.054	0.059	−2	96.7	−0.84	0.402
Health insurance	U	0.903	0.927	−8.5		−3.33	0.001
	M	0.897	0.909	−4.1	52	−1.37	0.171
Public pension	U	0.558	0.609	−10.3		−4.05	0.000
	M	0.533	0.512	4.1	60.4	1.44	0.151
Happiness	U	3.809	3.644	18.6		7.3	0.000
	M	3.776	3.782	−0.7	96.1	−0.27	0.789
Desired number of [illegible]	U	2.017	2.117	−16.3		−6.44	0.000
	M	2.037	2.027	1.6	90.2	0.61	0.545
Household income	U	10.869	10.113	78.4		30.89	0.000
	M	10.738	10.725	1.4	98.2	0.55	0.581
Childcare facilities	U	0.651	0.450	41.3		16.24	0.000
	M	0.618	0.612	1.1	97.4	0.38	0.705
Health clinics	U	0.866	0.855	3.4		1.34	0.181
	M	0.867	0.863	1	71.5	0.35	0.726
Sample	Ps R2	LR chi2	p>chi2	MeanBias	MedBias	B	R
Unmatched	0.286	2452.03	0.000	34.1	23.8	132.3*	2.49*
Matched	0.003	18.44	0.187	2.5	1.7	12.1	1.05

Notes: B represents absolute standard deviation and R denotes standard deviation ratio.

Table A2 The first stage of 2SLS regression

Variables	Off-farm employment participation
Number of enterprises	0.005***
	(0.002)
Distance to the nearest county	−0.074***
	(0.007)
Age	−0.009***
	(0.001)
Education	0.040***
	(0.003)
Religion	0.059***
	(0.016)
Ethnic minority	−0.175***
	(0.018)
Good health	0.027**
	(0.011)
Hospitalization	−0.025
	(0.021)
Maternity insurance	0.218***
	(0.015)
Health insurance	−0.061***
	(0.020)
Public pension	−0.072***
	(0.011)
Happiness	−0.008
	(0.006)
Desired number of children	0.016*
	(0.009)
Household income	0.101***
	(0.007)
Childcare facilities	0.133***
	(0.011)
Health clinics	−0.076***
	(0.015)
Constant	−0.235***
	(0.089)
Observations	6,188

Note: ***, **, and * indicate significance at the 1%, 5%, and 10% levels, respectively. Robust standard errors are presented in parentheses.
